# Development and evaluation of regression tree models for predicting in-hospital mortality of a national registry of COVID-19 patients over six pandemic surges

**DOI:** 10.1186/s12911-023-02401-2

**Published:** 2024-01-02

**Authors:** M. C. Schut, D. A. Dongelmans, D. W. de Lange, S. Brinkman, Dutch COVID-19 Research Consortium, D. P. Verbiest, L. F. te Velde, E. M. van Driel, T. Rijpstra, P. H. J. Elbers, A. P. I. Houwink, L. Georgieva, E. Verweij, R. M. de Jong, F. M. van Iersel, T. J. J. Koning, E. Rengers, N. Kusadasi, M. L. Erkamp, R. van den Berg, C. J. M. G. Jacobs, J. L. Epker, A. A. Rijkeboer, M. T. de Bruin, P. Spronk, A. Draisma, D. J. Versluis, A. E. van den Berg, M. Vrolijk-de Mos, J. A. Lens, R. V. Pruijsten, H. Kieft, J. Rozendaal, F. Nooteboom, D. P. Boer, I. T. A. Janssen, L. van Gulik, M. P. Koetsier, V. M. Silderhuis, R. M. Schnabel, I. Drogt, W. de Ruijter, R. J. Bosman, T. Frenzel, L. C. Urlings-Strop, A. Dijkhuizen, I. Z. Hené, A. R. de Meijer, J. W. M. Holtkamp, N. Postma, A. J. G. H. Bindels, R. M. J. Wesselink, E. R. van Slobbe-Bijlsma, P. H. J. van der Voort, B. J. W. Eikemans, D. Mehagnoul-Schipper, D. Gommers, J. G. Lutisan, M. Hoeksema, M. G. W. Barnas, B. Festen-Spanjer, M. van Lieshout, N. C. Gritters, M. van Tellingen, G. B. Brunnekreef, J. Vandeputte, T. P. J. Dormans, M. E. Hoogendoorn, M. de Graaff, D. Moolenaar, A. C. Reidinga, J. J. Spijkstra, R. de Waal, D. Ramnarain, D. W. de Lange, D. A. Dongelmans, N. F. de Keizer, A. Abu-Hanna

**Affiliations:** 1 Department of Medical Informatics, Amsterdam Public Health research institute, Amsterdam UMC location University of Amsterdam, Meibergdreef 9, 1105 AZ Amsterdam, The Netherlands; 2 Department of Laboratory Medicine, Amsterdam UMC location Vrije Universiteit, De Boelelaan 1117, 1081 HV Amsterdam, The Netherlands; 3 Department of Intensive Care Medicine, Amsterdam UMC location University of Amsterdam, Meibergdreef 9, 1105 AZ Amsterdam, The Netherlands; 4 Department of Intensive Care Medicine and Dutch Poisons Information Center (DPIC), University Medical Center Utrecht, University Utrecht, Heidelberglaan 100, 3584 CX Utrecht, The Netherlands

**Keywords:** Decision rules, Decision and regression trees, Prediction model, Intensive care, COVID-19, In-hospital mortality, Registry, Observational data

## Abstract

**Background:**

Objective prognostic information is essential for good clinical decision making. In case of unknown diseases, scarcity of evidence and limited tacit knowledge prevent obtaining this information. Prediction models can be useful, but need to be not only evaluated on how well they predict, but also how stable these models are under fast changing circumstances with respect to development of the disease and the corresponding clinical response. This study aims to provide interpretable and actionable insights, particularly for clinicians. We developed and evaluated two regression tree predictive models for in-hospital mortality of COVID-19 patient at admission and 24 hours (24 h) after admission, using a national registry. We performed a retrospective analysis of observational routinely collected data.

**Methods:**

Two regression tree models were developed for admission and 24 h after admission. The complexity of the trees was managed via cross validation to prevent overfitting. The predictive ability of the model was assessed via bootstrapping using the Area under the Receiver-Operating-Characteristic curve, Brier score and calibration curves. The tree models were assessed on the stability of their probabilities and predictive ability, on the selected variables, and compared to a full-fledged logistic regression model that uses variable selection and variable transformations using splines. Participants included COVID-19 patients from all ICUs participating in the Dutch National Intensive Care Evaluation (NICE) registry, who were admitted at the ICU between February 27, 2020, and November 23, 2021. From the NICE registry, we included concerned demographic data, minimum and maximum values of physiological data in the first 24 h of ICU admission and diagnoses (reason for admission as well as comorbidities) for model development. The main outcome measure was in-hospital mortality. We additionally analysed the Length-of-Stay (LoS) per patient subgroup per survival status.

**Results:**

A total of 13,369 confirmed COVID-19 patients from 70 ICUs were included (with mortality rate of 28%). The optimism-corrected AUROC of the admission tree (with seven paths) was 0.72 (95% CI: 0.71–0.74) and of the 24 h tree (with 11 paths) was 0.74 (0.74–0.77). Both regression trees yielded good calibration and variable selection for both trees was stable. Patient subgroups comprising the tree paths had comparable survival probabilities as the full-fledged logistic regression model, survival probabilities were stable over six COVID-19 surges, and subgroups were shown to have added predictive value over the individual patient variables.

**Conclusions:**

We developed and evaluated regression trees, which operate at par with a carefully crafted logistic regression model. The trees consist of homogenous subgroups of patients that are described by simple interpretable constraints on patient characteristics thereby facilitating shared decision-making.

**Supplementary Information:**

The online version contains supplementary material available at 10.1186/s12911-023-02401-2.

## Background

Objective prognostic information describing the probability of survival for patients may inform intensivists in improving triage before the admission and proportionality of treatment during the admission [1]. The time-limited trial (TLT) has been proposed as a practical approach to establish the goals of intensive care unit (ICU) care when the benefits of ICU interventions are uncertain [2]. This seemed especially relevant during the pandemic COVID-19 surges when ICU resources were scarce [3].

When objective prognostic information is not available, intensivists can only estimate survival probabilities based on scarcely available evidence, as well as on tacit knowledge relying on variables such as age, the number and severity of comorbidities, and frailty, which are based on experiences from the past. In case of a pandemic with an unknown disease, it is not known if general knowledge, for instance based on age is still valid. Even more so, since the pandemic rapidly evolved (fast development of different variants of the virus, and introduction and fast development of new treatment guidelines and vaccination), it is also unknown how stable the scarce objective prognostic information is over time.

Objective prognostic information can be obtained with prediction models. Such models vary from relatively simple models that are easily interpretable (e.g., decision and regression trees) to complex but often hard-to-interpret models (e.g., neural networks). This study aims to provide interpretable and actionable insights, particularly for clinicians. This immediate clinical application of our study required that the developed models must be easily interpretable, used only variables that are robust and are in the eyes of clinicians understandable, and did not require a computer to provide the predictions. This has led us to rely on a simple tree-based prediction approach that provides predictions and insights into high-risk groups [4]. A tree model can also be used to define patient subgroups corresponding to the tree paths (i.e., from tree root to each leaf) and containing those patients fulfilling the conditions (e.g., on age, number of comorbidities, APACHE score, etcetera) on that path.

We aimed to assess in-hospital survival probabilities of COVID-19 patients admitted to the ICU by using regression trees and inspected the corresponding COVID-19 subgroups. Additionally, we analysed (1) the Length-of-Stay (LoS) per subgroup per survival status, and (2) the stability of the discovered subgroups in terms of the survival probabilities over the six different COVID-19 surges in The Netherlands.

## Methods

### Population

This study used prospectively collected data on patients admitted between February 27 2020 and November 23, 2021 with confirmed COVID-19 to Dutch ICUs, extracted from the Dutch National Intensive Care Evaluation (NICE) registry [5]. The NICE dataset contains, amongst other items, demographic data, minimum and maximum values of physiological data in the first 24 h of ICU admission, diagnoses (reason for admission as well as comorbidities), ICU as well as in-hospital mortality data and length of stay. The data collection takes place in a standardized manner according to strict definitions and stringent data quality checks to ensure high data quality. The registry did not include information on COVID-19 vaccination status. The (number of) chronic comorbidities include: (1) immunological insufficiency or AIDS; (2) chronic renal failure or chronic dialysis; (3) respiratory failure or COPD; (4) neoplasm or hematologic malignancy; (5) cardiovascular insufficiency; (6) liver cirrhosis; (7) diabetes. We included patient data that were available within the first 24 h after admission.

### Patient inclusion

Patients were considered to have COVID-19 when either the RT-PCR of their respiratory secretions was positive for SARS-CoV-2, or (during the early surges when RT-PCR tests had limited availability), when their CT-scan was consistent with COVID-19 (i.e. a CO-RADS score of ≥4 in combination with the absence of an alternative diagnosis) [6].

### Outcome measurements

The primary outcome of this study was in-hospital mortality. During the peaks of COVID-19 there was a shortage of ICU beds in some hospitals and many patients were transferred to other ICUs. For transferred patients we could follow their transfers through the Netherlands or Germany (because all Dutch ICUs participate in the used registry) and used the survival status of the last hospital the patient was admitted to during one and the same COVID-19 episode. Additionally, we analysed as secondary outcome the hospital and ICU Length-of-Stay (LoS) per subgroup per survival status.

### Model development

Decision and regression tree-based models are easily interpretable, non-parametric prediction models. They allow for complex non-linear relationships between predictors and outcomes, can handle missing values [7], and variable selection forms part of the learning algorithm. We developed two regression tree models, one with only admission data and one with first 24-hours (24 h) data. Supplementary Table S7 includes an overview of the used variables contained in the admission and 24 h datasets, respectively. We relied on the regression tree mechanism to impute missing values, and hence no imputation was attempted beforehand. The tree consists of nodes (patient subgroups) and branches (constraints on variable values). The entire patient group forms the top node and is iteratively split into two nodes (subgroups) until a predefined stopping criterion, e.g., depth of the tree or minimal subgroup size. The further working of decision trees is explained in Supplementary Text S1. Throughout the paper, we use the term subgroup to denote a path in the tree from top (root) to bottom (leaf), corresponding with a group of patients that fulfil the conditions on that path. The subgroup is associated with a survival probability (this is the proportion of survivors within the group). To avoid overfitting, the complexity of the tree (i.e., its depth and hence its size) was determined by finding the complexity that corresponds to the minimal 10-fold cross-validated error plus its standard deviation, akin to what we did in [4]. This complexity was then used to fit the final tree on the whole dataset. Limiting the tree’s complexity also means that not all available variables need to be chosen in the tree.

### Model evaluation

We used bootstrapping to evaluate the predictive performance of both trees on discrimination using the Area under the Receiver-Operating-Characteristic curve, (AUROC) curve, and on prediction accuracy by means of the Brier score (i.e., the mean squared error of the prediction). We also inspected calibration with calibration curves. Internal model validation of the AUROC and Brier measures was based on 200 bootstrap samples and 95% confidence intervals were computed from the resampling distribution with percentile intervals. Calibration curves were based on 10-fold cross validation.

We did post hoc validation analyses in which we tested both trees on a dataset including patients up to January 1st, 2023.

### Stability of variables selected in the tree

In the 200 bootstrap samples of the model development procedure, we counted the times that a variable was selected in the corresponding bootstrap trees. We then compared the resulting variable frequency with variables appearing in our final trees.

### Subgroup analysis

To get insight into the behaviour of subgroups we performed the following calculations on them:

1. We analysed the Length-of-Stay (LoS) per patient subgroup per survival status for ICU and hospital, because ICU LoS of COVID-19 patients tends to be generally higher, and longer treatment time is acceptable if survival chances are sufficiently high.
2. Although regression trees may be easily interpretable, they may not be stable over time. Therefore, we computed and compared the survival probabilities for each patient subgroup for each COVID-19 surge. Table 1 overviews the time periods of the COVID-19 surges in The Netherlands.
3. Since regression trees may not perform as well as standard crafted parametric regression models and logistic regression is the most commonly used prediction model in intensive care, we developed a full-fledged logistic regression (LR) model and compared probabilities of the regression tree with the predictions of the LR model. Parameters used for both trees and logistic regression were kept the same, e.g., the model was trained with the same data, and the same performance measures were used. For this analysis we used one imputation using chained eqs [8]. When the continuous variables had a non-monotonic association with the outcome they were transformed by restricted cubic splines. The best predictors were selected with a backward AIC (Akaike Information Criterion) variable selection procedure. We obtained per patient subgroup the mean, median, and 1st and 3rd quartiles of the LR predictions.
4. We assessed the added predictive performance of subgroups. Hereto we added a new variable to the LR model described above indicating the subgroups to which a patient belongs. We performed variable selection using the AIC to inspect whether this variable would survive the variable selection. In addition, we used ANOVA to test whether the LR model with the subgroup variable was significantly better than without the variable.

**Table 1:** Overview of dates for the COVID-19 surges in The Netherlands

Surge	From	To
1	1 February 2020	15 May 2020
0 (in between)	16 May 2020	30 September 2020
2	1 October 2020	1 December 2020
3	1 December 2020	31 January 2021
4	1 February 2021	1 July 2021
5	1 July 2021	30 September 2021
6	1 October 2021	23 November 2021

### Statistical analysis

To analyse the data, we used the statistical environment R version 3.6.1. We used the MICE package for imputation, the MASS package for stepwise model selection, and the Rpart package for recursive partitioning (decision tree). The R packages are available for download from https://cran.r-project.org/web/packages/.

## Results

### Study population

A total of 13,369 confirmed COVID-19 patients from 70 ICUs were included; 3726 patients (28%) died during their hospital stay. Table 2 shows the descriptive summary statistics of the patient population stratified by hospital survival state. Compared to survivors, the non-survivors were older (69 vs 60 years, *p* < 0.001) more often male (73.5% vs 67.3%, *p* < 0.001), had more comorbidities and a much higher APACHE II score (73 vs 57 points, *p* < 0.001).

**Table 2:** Descriptive statistics of baseline characteristics of the study population stratified by hospital survival state

	Survived hospitalization	Deceased during hospitalization	*P*-value
Number of patients	9643	3726	
Age (mean (SD))	59.9 (12.1)	68.59 (9.0)	< 0.001
Male (%)	6486 (67.3)	2740 (73.5)	< 0.001
BMI (mean(SD))	29.8 (5.6)	28.96 (5.5)	< 0.001
Chronic diagnosis			
Respiratoir insufficiency	1097 (11.4)	710 (19.1)	< 0.001
Renal insufficiency	270 (2.8)	347 (9.3)	< 0.001
Chirrosis	25 (0.3)	32 (0.9)	< 0.001
Malignancy	171 (1.8)	64.8 (1.7)	< 0.001
Immunological insufficiency	714 (7.4)	524 (14.1)	
Cardiovasculair insufficiency	103 (1.1)	105 (2.8)	< 0.001
Number of comorbidities (mean (SD))	0.44 (0.7)	0.76 (0.9)	< 0.001
Admission type N(%)			0.31
Medical	9457 (98.3)	3668 (98.7)	
Urgent surgery	114 (1.2)	31 (0.8)	
Elective surgery	49 (0.5)	18 (0.5)	
Missing admission type or died before ICU admission	23 (3.6)	9 (0.2)	
Mechanical ventilated in first 24 hrs of ICU admission	5672 (58.8)	2638 (70.8)	< 0.001
APACHE III score	56.57 (18.3)	72.52 (23.8)	< 0.001
ICU mortality N(%)			
In-hospital mortality N(%)	0 (0)	3726 (100)	

### Model development

**Fig. 1:**
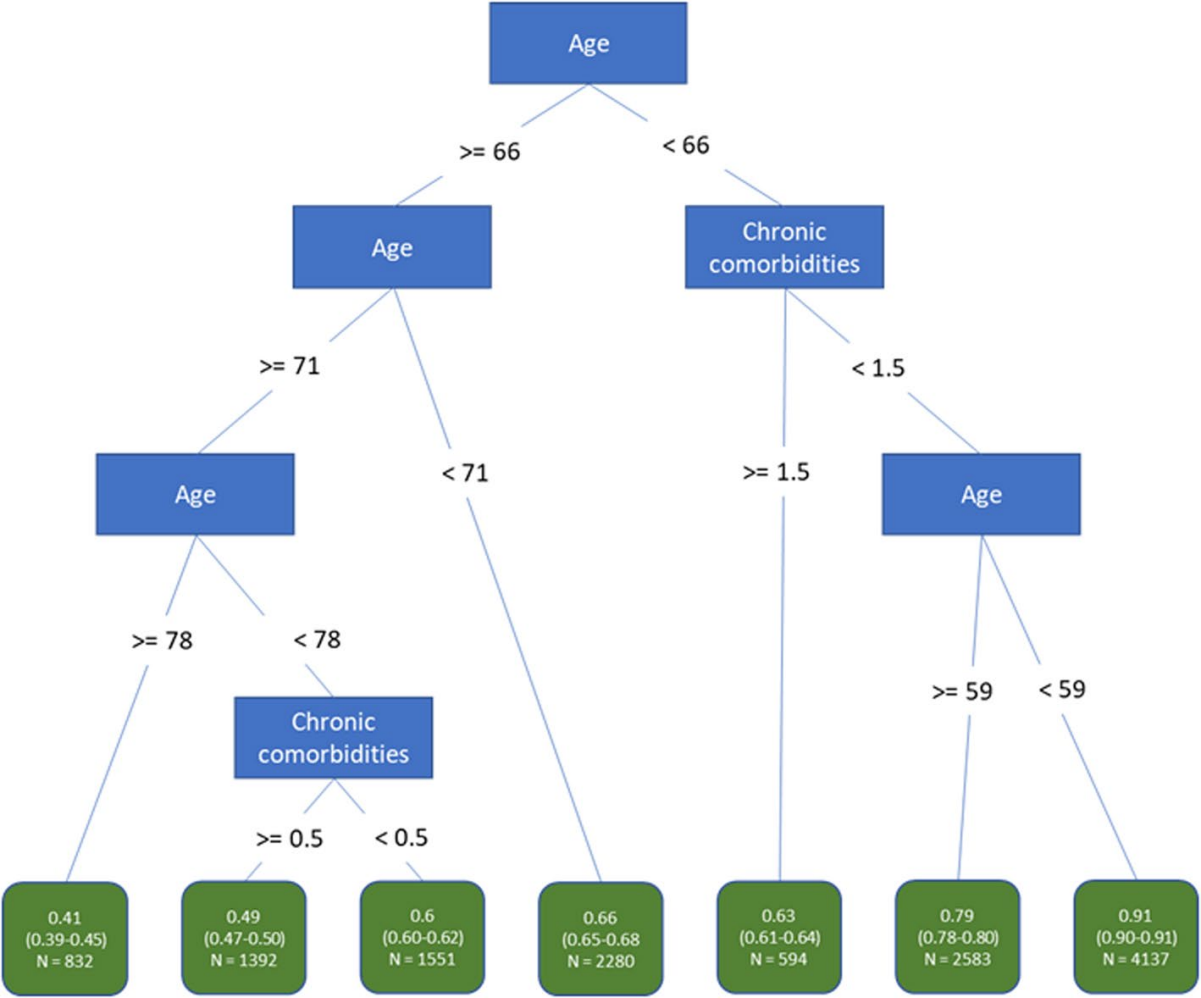
Regression tree for survival (#ch.com is the number of chronic comorbidities) based on patient data available at admission. Each node shows the survival probabilities in that group as well as the number of patients in that group. Survival probabilities include a 95% confidence interval based on the percentile bootstrap method on 200 samples

Figure 1 shows the learned regression tree based on data that were available at admission. We found that two variables (age and number of chronic comorbidities) were selected, and that age is at the root (top) of the tree, which can be understood as the most important variable in the population. Patients from 78 years on have the worst chance to survive (41%). Patients younger than 59 years and with at most one comorbidity have the best chance to survive (91%). These worst and best survival probabilities differ considerably from the population survival average of 72%.

**Fig. 2:**
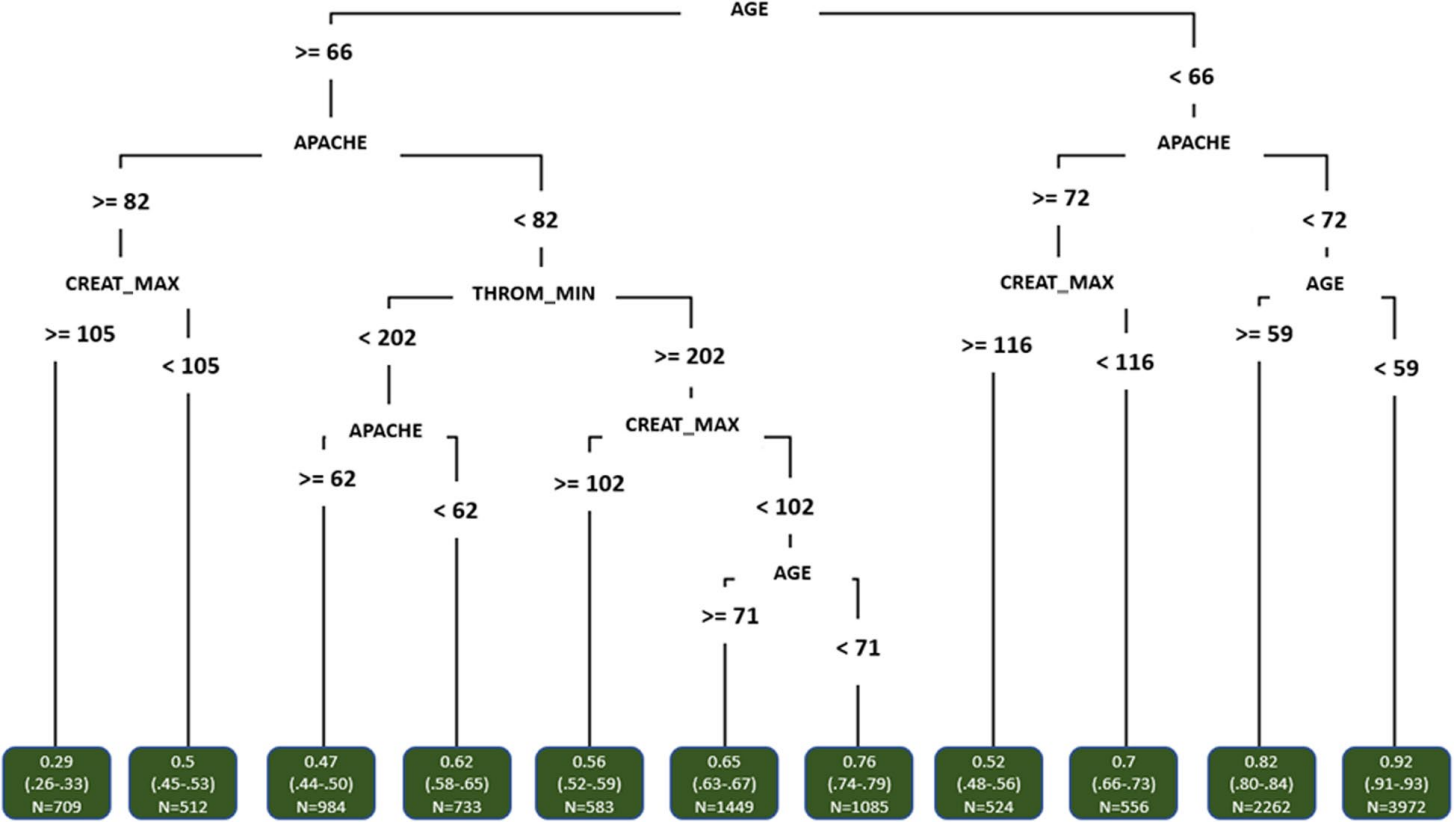
Regression tree for survival based on patient data available 24 h after admission. Each node shows the survival probabilities in that group as well as the number of patients in that group. Survival probabilities include a 95% confidence interval based on the percentile bootstrap method on 200 samples

The regression tree based on data that were available 24 h after admission is included in Fig. 2. We observe that the 24 h tree is more specific because it contains more complex subgroups; age is included in both trees, but the number of comorbidities is not; and in the 24 h tree the APACHE score and maximum creatinine level (both not available at admission) are included as most important variables after age.

### Model evaluation

**Fig. 3:**
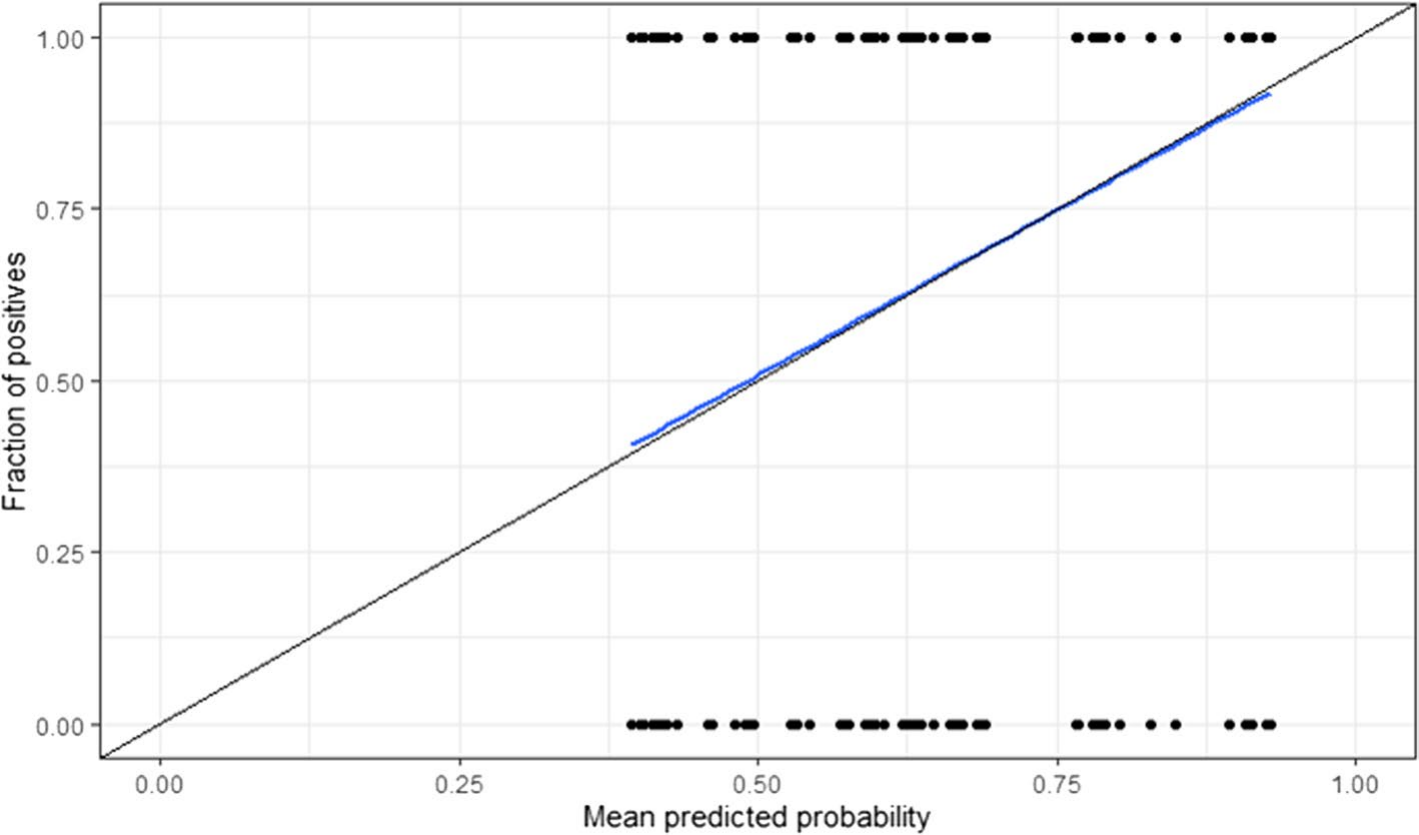
Calibration curve for the regression tree based on patient data available at admission

The optimism-corrected AUROC of the admission tree was 0.72 (95%CI: 0.71–0.74), which means the model yielded acceptable discrimination. The admission tree yielded a Brier score of 0.175 (0.171–0.179). The calibration curve for the admission tree is included in Fig. 3, showing good calibration (curve closely follows the ideal curve).

For the 24 h tree, the optimism corrected AUROC was 0.74 (0.74–0.77), which means that also this model yielded fair discrimination. The 24 h tree yielded a Brier score of 0.168 (0.163–0.171), which is low and thus indicative of good calibration. The calibration curve for the 24 h tree is included in Supplementary Figure s1, also showing good calibration.

In the post hoc validation analyses, the admission tree achieved an AUROC of 0.71 and for the 24 h tree, the AUROC was 0.75.

### Stability of variables selected in the tree

For both the admission and 24 h trees, variable frequencies over the bootstrap samples are included in Table 3 and Supplementary Table s4, respectively. The variables in the trees (0 h: patient age, number of chronic comorbidities; 24 h: patient age, APACHE score, and others) were selected in most samples. The next most frequently included variables were selected with a much lower frequency.

**Table 3:** Selected variables in number of bootstraps. Analysed model was based on admission data

Variable	Frequency
Age	200
Number of comorbidities	198
Mechanical ventilated at admission	78
Surge	18
Gender	17
Admission source	10
Immunological Insufficiency	7
BMI	6
Hospital length of stay before ICU admission	5
Referring specialty	3

### Subgroup analysis

1. The average length of stay (LoS, in days) is shown in Table 4 (0 h) and Supplementary Table s3 (24h) per patient subgroup per survival status for ICU and hospital, respectively. (For convenience of referral, subgroups are numbered by tree leaf from left to right in the regression trees; see also Supplementary Table s1 and Supplementary Table s2). For the admission tree, we see that for surviving patients, subgroup 1 has the shortest ICU LoS (11.7 days) and subgroup 2 has the longest ICU LoS (20.6 days). For non-surviving patients, these are subgroup 1 (shortest ICU LoS: 12.3 days) and 6 (longest ICU LoS: 18.4 days), respectively. In general, ICU LoS varies between 11 and 25 days, and hospital stay is at least 4 days and at most 25 days after ICU discharge.

**Table 4:**
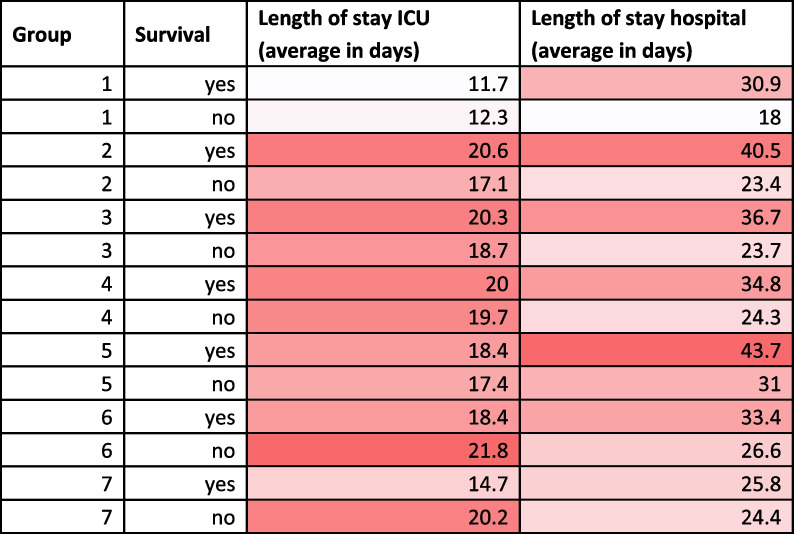
Average Length-of-Stay (LoS, in days) in ICU and hospital per group per survival status. The hue is relative per column and is from shortest stay (white) to longest stay (red). Analysed model was based on admission data
2. Within each leaf (patient subgroup), each COVID surge had a survival probability that was generally comparable to the survival probability in the leaf as a whole. Still, for leaves with smaller subgroup sizes, there was a larger variation in probabilities over the surges. The survival probabilities over all surges are included in Table 5 and Supplementary Table s5.
3. The 0 h and 24 h logistic regression (LR) models are reported in two other articles. The LR model at admission (0 h) is included in Supplementary Table 4 of [9] and the LR model at 24 h is included in Supplementary Table s2 of [10]. The probabilities of the regression tree are in good agreement with the means and medians of the predictions of the LR model. The LR model survival probabilities for all subgroups are included in Table 6 and Supplementary Table s6.
4. For both admission and 24 h data, the models with the ‘subgroup’ variable were significantly better than the models without this variable. Also, this ‘subgroup’ variable was selected after backward selection based on the AIC. This means that the subgroups have additional predictive value on the logistic regression model without this variable.

**Table 5:** Survival probabilities over all surges. Analysed model was based on admission data

Group	Surge							Average	Stdev
	0	1	2	3	4	5	6		
1	0.49	0.28	0.37	0.47	0.44	0.68	0.42	0.45	0.12
2	0.42	0.49	0.49	0.42	0.52	0.57	0.55	0.49	0.06
3	0.57	0.59	0.62	0.55	0.64	0.55	0.52	0.58	0.04
4	0.69	0.64	0.63	0.64	0.71	0.65	0.58	0.65	0.04
5	0.59	0.67	0.61	0.55	0.68	0.60	0.54	0.60	0.05
6	0.77	0.77	0.78	0.80	0.80	0.79	0.67	0.77	0.05
7	0.93	0.89	0.89	0.90	0.92	0.93	0.94	0.91	0.02

**Table 6:** Variance of the survival chances in the leaves of the tree according to the best logistic regression (LR) model. Analysed model was based on admission data

Group	N	Survival chance in group (%)	Average LR model prediction in group (%)	Median LM model prediction and 1st and 3d quantiles (%)
1	832	41.2	42.4	44.8 [36.2–52.5]
2	1392	48.6	49.2	51.0 [42.1–58.6]
3	1551	60.3	61.7	62.6 [57.4–67.1]
4	2280	66.4	66.3	69.4 [61.9–74.1]
5	594	63	67.5	68.7 [59–77.7]
6	2583	78.8	77.8	79.3 [74.5–82.9]
7	4137	91	90.1	90.7 [86.8–94.5]

## Discussion

### Findings

In our analyses we used regression trees to show combinations of predictors that affect the patient’s survival chances. These trees show that age in isolation is a poor prognosticator of in-hospital mortality and is only useful in very advanced age (age > = 78 years). However, the combination of age in combination with chronic comorbidities does have a clear prognostic value. The admission tree shows that for patients aged > = 59, the number of comorbidities has a major impact on the survival chances: with one comorbidity, the survival probability is 0.79 but with multiple (> = 2) comorbidities, survival chances drop to 0.63. On the other hand, the 24 h tree shows that patients aged < 59 with an APACHE score < 72, have the highest survival probability (.92).

Regarding ICU and hospital length-of-stay (LoS), COVID-19 patients tend to stay long in ICU [11]. Such longer treatment time is deemed acceptable if the chances on patient survival are higher. We analysed the ICU and hospital LoS per patient subgroup per survival status and confirmed these rather long ICU stays: the average ICU LoS was 18 days and the average hospital LoS was 30 days. However, we could not discern a particular subgroup with an exceptionally short/long ICU/hospital LoS.

Analysing the regression tree model over six (chronological) COVID-19 surges over a period of 1.5 years showed that the survival probabilities of the subgroups were generally stable. We only observed larger probability variations for smaller subgroups. This stability means that despite the changes in triage decisions, treatments and developments of COVID-19 itself in the analysed time period, the regression tree models were not very sensitive to such dynamics [12].

As for the potential effectiveness of time-limited trial (TLT) admissions, we did obtain a regression tree when using data available at 24 h after admission that was more pronounced than when using data available at admission, but the difference in predictive performance between these trees was small and expectedly not clinically relevant. Still, the 24 h regression tree itself showed selection of the most informative predictors (i.e., age, APACHE score, highest creatinine value, lowest platelets count) and combinations of these predictors.

### Related work

Many prognostic models of mortality among COVID-19 infected patients have been published, and an extensive overview of such model studies is given in [13]. The overview shows that the predictive performance of these models is fair to excellent, but many studies lack temporal validation. An exception to this is [14] that does perform temporal model validation (albeit not over different surges but more traditional in the sense of using different time periods for model training and testing, respectively), but it has a relatively small population size (*n* = 679) and the timing of the prediction is on day 7 after admission, which is a later-in-disease-course-prediction than in our study. We did validate our models extensively over the different COVID-19 surges. And while there have been (national) initiatives to set up surveillance platforms to identify temporal trends and COVID-19 risk factors with patient-level data, e.g., [15], these studies tend to focus on trends in descriptive data and on the mere impromptu design and setup of such platforms. We looked at temporal validation of predictive models and used a quality registry that has already been in existence for over 25 years [5].

Other studies have also used machine learning algorithms for predicting ICU admission and mortality among COVID-19 patients. So did [16] compare the performance of 18 machine learning algorithms and [9] used automated machine learning with 20 algorithms for ICU triage prediction of in-hospital mortality of COVID-19 patients. Still, since our objective was to deliver simple interpretable models that could be easily used in practice, more advanced machine learning methods were not in scope for this study.

Regarding the development of such practical models, there still have been several studies with similar aims. In [17] simple and valid models were developed for predicting mortality and need for intensive care unit (ICU) admission in patients who present at the emergency department (ED) with suspected COVID-19; however, their use of regression models prevents the straightforward and automatic discovery of combinations of predictors like our decision tree model does. Likewise, in [18] a pragmatic risk score is developed and validated to predict mortality in patients admitted to hospital with COVID-19. Still, the study includes (tree-based) machine learning methods to account for complex underlying interactions. However, the models have not been temporally validated, like we do over different COVID-19 surges. Finally, one study [19] used machine learning on routinely collected clinical data and assess temporal patterns with a methodology that was very comparable to ours. However, they aimed at identifying biologically distinct sub-phenotypes while we focused on patient characteristics.

In this work we focused on regression trees because the aim and scope of this study is to provide insight into the task at hand, and to comply with the stated model requirements. If, however, performance was the most important requirement then non-parametric models such as random forests and XGB models could be investigated. However, these models would require a computer to provide the predictions.

### Strengths

We developed simple but effective tree models that deliver transparent decision rules that could be used for triage purposes. The dataset that we used was extensive with national coverage of over almost 2 years.

We used regression trees that predict the probability of an event which are more useful than trees that deterministically predict the event (i.e., classification), because deterministic predictions communicate a single outcome but with no information about risk.

### Limitations

This study has some limitations. Firstly, the used registry data did not contain certain information that may have been relevant. We had no information on vaccination status, which could have influenced the stability of the tree at the later surges. We also did not include information on the COVID-19 virus variants, e.g., alpha, delta and omicron. Secondly, we did not have access to data on quality of life (QoL) in the survivors while this outcome measure is of utmost importance. For critically ill old intensive care patients, the quantification of health related QoL as suggested by [20] can be used on admission to inform patients of unfavourable qualitative outcomes if such patients survive. Finally, a limitation of trees is that all rules use the same variable that is in the top of the tree. We used the following analysis to give a chance to other variables to be selected (results not shown). After learning the original tree, we removed the data of the patients in the highest risk group and learned another tree on the remainder of the data. We repeated this procedure and upon completion of learning the trees, we identified the patient subgroups from all trees and compared these groups with the groups of the original tree. This analysis resulted in a series of trees that together had the same groups as our original tree, hence the impact of this limitation is small.

### Implications

The simple-to-interpret regression trees seem to be valuable in discussions with patients and their family. This study showed that these models remain stable over time which is an important feature. The results may also be useful for triage, but not solely based on this study: further study on validation, robustness, feasibility, impact et cetera is needed then.

### Future work

Further and continuous development and validation of the regression trees (and alternative models like Random Forests and eXtreme Gradient Boosting models) form future work. Likewise, different disease variations, changes in treatment and/or triage, and the availability of more patient characteristics, e.g., vaccination status, will affect the trees and their predictive performance and require model revalidation. Also, in case of future COVID-19 surges, the trees need to be validated to confirm the model stability we found over the past six surges.

### Conclusion

We used registry patient data to develop and evaluate two regression tree predictive models for in-hospital survival of COVID-19 patient at admission and 24 h after admission. The trees achieved acceptable predictive performance (fair discrimination, good accuracy and calibration, on par with full-fledged logistic regression models) and were generally stable (in terms of selected variables and over the COVID-19 surges). We analysed the regression trees to obtain subgroups of patients that have the same survival chances, and which are described by constraints on the predictors (patient characteristics) and that can be interpreted as clinical decision rules and used in shared decision making.

### Supplementary Information

**Additional file 1.**

**Additional file 2.**

## Data Availability

The data that support the findings of this study are available from NICE but restrictions apply to the availability of these data, which were used under license for the current study, and so are not publicly available. Data are however available from the authors upon reasonable request and with permission of NICE through https://www.stichting-nice.nl/extractieverzoek_procedure.jsp.

## Declarations

### Compliance

All methods were carried out in accordance with relevant guidelines and regulations.
