# Supplementary material for: Development and evaluation of regression tree models for predicting in-hospital mortality of a national registry of COVID-19 patients over six pandemic surges
Source: BMC Med Inform Decis Mak. 2024 Jan 2;24:7. doi: 10.1186/s12911-023-02401-2 (PMC10762959; doi:10.1186/s12911-023-02401-2)
Supplement: Supplementary file 1 — Additional file 1. [file 12911_2023_2401_MOESM1_ESM.docx]

**Supplementary materials**

< Figure-s1.tif>

**Supplementary Figure s1.** Calibration curve for the regression tree based on patient data available 24 hours after admission.

| **Group** | **N (% of dataset)** | **Rule condition(s)** | **Rule consequence** |
| --- | --- | --- | --- |
| 1 | 832 (6) | IF age ≥ 78 | THEN survival = 41.2% |
| 2 | 1,392 (10) | IF 71 ≤ age ≤ 77 AND #com ≥ 1 | THEN survival = 48.6% |
| 3 | 1,551 (12) | IF 71 ≤ age ≤ 77 AND #com =0 | THEN survival = 60.3% |
| 4 | 2,280 (17) | IF 66 ≤ age ≤ 71 | THEN survival = 66.4% |
| 5 | 594 (4) | IF age ≤ 65 AND #com >= 2 | THEN survival = 62.5% |
| 6 | 2,583 (19) | IF 59 ≤ age ≤ 65 AND #com ≤ 1 | THEN survival = 78.7% |
| 7 | 4,137 (31) | IF age ≤ 58 AND #com ≤ 1 | THEN survival = 91% |

**Supplementary Table s1.** Decision rules of the regression tree (#com is the number of chronic comorbidities), where each rule corresponds with a tree path (going from the tree root to a leaf). Analysed model was based on admission data.

| **Group** | **N (% of dataset)** | **Rule condition(s)** | **Rule consequence** |
| --- | --- | --- | --- |
| 1 | 709 (5) | IF age ≥ 66 AND apache ≥ 82 AND  creat_max ≥ 105 | THEN survival = 29% |
| 2 | 512 (4) | IF age ≥ 66 AND apache ≥ 82 AND  creat_max < 105 | THEN survival = 50% |
| 3 | 984 (7) | IF age ≥ 66 AND 62 ≤ apache < 82 AND  throm_min < 202 | THEN survival = 47% |
| 4 | 733 (5) | IF age ≥ 66 AND apache < 62 AND  throm_min < 202 | THEN survival = 62% |
| 5 | 583 (4) | IF age ≥ 66 AND apache < 82 AND  throm_min ≥ 202 AND  creat_max ≥ 102 | THEN survival = 56% |
| 6 | 1449 (11) | IF age ≥ 71 AND apache < 82 AND  throm_min >= 202 AND  creat_max < 102 | THEN survival = 65% |
| 7 | 1085 (8) | IF 66 ≤ age < 71 AND apache < 82 AND  throm_min >= 202 AND  creat_max < 102 | THEN survival = 76% |
| 8 | 524 (4) | IF age < 66 AND apache ≥ 72 AND  creat_max ≥ 116 | THEN survival = 52% |
| 9 | 556 (4) | IF age < 66 AND apache ≥ 72 AND  creat_max < 116 | THEN survival = 70% |
| 10 | 2262 (17) | IF 59 ≤ age < 66 AND apache < 72 | THEN survival = 82% |
| 11 | 3972 (30) | IF age < 59 AND apache < 72 | THEN survival = 92.1% |

**Supplementary Table s2.** Decision rules of the regression tree (creat_max is the highest serum creatinine value in the first 24 hours of ICU admission; throm_min is the lowest value for platelets in the first 24 hours of ICU admission), where each rule corresponds with a tree path (going from the tree root to a leaf). Analysed model was based on 24h data.

| **Group** | **Survival** | **Lenth of stay ICU** | **Length of stay hospital** |
| --- | --- | --- | --- |
|  |  | **(average in days)** | **(average in days)** |
| 1 | yes | 21.1 | 45 |
| 1 | no | 12.8 | 17.3 |
| 2 | yes | 20.9 | 38.4 |
| 2 | no | 16.9 | 22.4 |
| 3 | yes | 23.5 | 42.5 |
| 3 | no | 18.2 | 23.6 |
| 4 | yes | 20.8 | 35.7 |
| 4 | no | 19.7 | 24.7 |
| 5 | yes | 20.2 | 40.1 |
| 5 | no | 17.4 | 22.4 |
| 6 | yes | 16.8 | 33.2 |
| 6 | no | 18.5 | 25.3 |
| 7 | yes | 18.1 | 31.4 |
| 7 | no | 19.9 | 24.5 |
| 8 | yes | 22.6 | 43.2 |
| 8 | no | 14.3 | 20.2 |
| 9 | yes | 19.9 | 38.6 |
| 9 | no | 17.6 | 27.9 |
| 10 | yes | 17.7 | 32.4 |
| 10 | no | 23.3 | 27.9 |
| 11 | yes | 14.5 | 25.8 |
| 11 | no | 23 | 29.8 |

**Supplementary Table s3.** Average Length-of-Stay (LoS, in days) in ICU and hospital per group per survival status. The hue is relative per column and is from shortest stay (white) to longest stay (red). Analysed model was based on 24h data.

| **Variabele** | **Frequency** |
| --- | --- |
| Age | 200 |
| APACHE | 200 |
| Lowest value for platelets  in the first 24 hours of ICU admission | 195 |
| Highest serum creatinine value  in the first 24 hours of ICU admission | 163 |
| Arterial pH | 49 |
| Lowest bicarbonate  in the first 24 hours of ICU admission | 37 |
| Highest serum urea value  in the first 24 hours of ICU admission | 25 |
| Mechanical ventilated  in first 24 hrs of ICU admission | 22 |
| Lowest serum creatinine value  in the first 24 hours of ICU admission | 21 |
| Highest heart rate  in the first 24 hours of ICU admission | 14 |
| Number of comorbidities | 12 |
| Lowest heart rate  in the first 24 hours of ICU admission | 11 |
| Highest bicarbonate  in the first 24 hours of ICU admission | 7 |
| Percentage of inhaled oxygen | 3 |
| Hospital length of stay before ICU admission | 3 |
| Vasoactive medication | 3 |
| Highest alveolar-arterial oxygen pressure difference | 2 |
| Lowest value of the ratio between PaO2 and FiO2  in the first 24 hours of ICU admission | 2 |
| Lowest systolic blood pressure | 2 |
| Highest body temperature | 2 |
| BMI | 1 |
| Lowest hematocrit | 1 |
| Arterial CO2 | 1 |
| Arterial oxygen pressure | 1 |
| Lowest respiratory rate | 1 |
| Lowest body temperature | 1 |

**Supplementary Table s4.** Selected variables in number of bootstraps. Analysed model was based on 24h data.

| **Group** | **Surge** |  |  |  |  |  |  | **Average** | **Stdev** |
| --- | --- | --- | --- | --- | --- | --- | --- | --- | --- |
|  | **0** | **1** | **2** | **3** | **4** | **5** | **6** |  |  |
| 1 | 0.33 | 0.29 | 0.22 | 0.26 | 0.38 | 0.25 | 0.07 | 0.26 | 0.10 |
| 2 | 0.59 | 0.38 | 0.45 | 0.49 | 0.57 | 0.58 | 0.64 | 0.53 | 0.09 |
| 3 | 0.44 | 0.55 | 0.42 | 0.42 | 0.50 | 0.39 | 0.27 | 0.43 | 0.09 |
| 4 | 0.60 | 0.63 | 0.63 | 0.51 | 0.66 | 0.67 | 0.38 | 0.58 | 0.10 |
| 5 | 0.56 | 0.48 | 0.54 | 0.54 | 0.61 | 0.78 | 0.67 | 0.59 | 0.10 |
| 6 | 0.55 | 0.63 | 0.64 | 0.66 | 0.65 | 0.72 | 0.81 | 0.67 | 0.08 |
| 7 | 0.84 | 0.71 | 0.73 | 0.75 | 0.80 | 0.82 | 0.71 | 0.77 | 0.05 |
| 8 | 0.26 | 0.47 | 0.53 | 0.53 | 0.59 | 0.49 | 0.11 | 0.43 | 0.17 |
| 9 | 0.77 | 0.68 | 0.68 | 0.68 | 0.72 | 0.75 | 0.29 | 0.65 | 0.17 |
| 10 | 0.88 | 0.80 | 0.81 | 0.83 | 0.83 | 0.82 | 0.79 | 0.82 | 0.03 |
| 11 | 0.93 | 0.91 | 0.91 | 0.91 | 0.93 | 0.94 | 0.96 | 0.93 | 0.02 |

**Supplementary Table s5.** Survival probabilities over all surges. Analysed model was based on 24h data.

| **Group** | **N** | **Survival chance in group (%)** | **Averge LR model prediction in group (%)** | **Median LM model prediction and 1st and 3d quantiles (%)** |
| --- | --- | --- | --- | --- |
| 1 | 709 | 28.9 | 31.3 | 29.1 [17.7-43.3] |
| 2 | 512 | 49.6 | 48 | 48.3 [34.7-61] |
| 3 | 984 | 47.1 | 50.7 | 51.4 [39.5- 62.2] |
| 4 | 733 | 61.7 | 62.1 | 63.5 [53.8-73] |
| 5 | 583 | 55.7 | 54.4 | 54.9 [44.1-66] |
| 6 | 1449 | 64.9 | 64.8 | 66.5 [56.6-74.7] |
| 7 | 1085 | 76.4 | 75.6 | 78.1 [69.4-83.6] |
| 8 | 524 | 52.3 | 55.7 | 57.7 [39.5-71.8] |
| 9 | 556 | 69.8 | 71.9 | 74.9 [61.6-85.3] |
| 10 | 2262 | 82 | 80.8 | 83.2 [76.3-88.4] |
| 11 | 3972 | 92.1 | 91.3 | 93.3 [88.2-96.5] |

**Supplementary Table s6.** Variance of the survival chances in the leaves of the tree according to the best logistic regression (LR) model. Analysed model was based on 24h data.

| **Variable** | Meaning | **0h** | **24h** |
| --- | --- | --- | --- |
| leeftijd | The patient’s age. | • | • |
| gender | The patient’s gender. | • | • |
| copd | Chronic Obstructive Pulmonary Disease | • | • |
| resp_insuf | Chronic restrictive, obstructive or vascular conditions in the lungs resulting in very severe restriction of mobility | • | • |
| chr_renal_insuf | If there is evidence of raised serum creatinine > 177 umol/L (2.0 mg/dl) and renal insufficiency in the medical history (before the current hospital admission) is classed as chronic. | • | • |
| chron_dialysis | The patient has been receiving long-term haemodialysis or peritoneal dialysis prior to the current hospital admission. | • | • |
| cirrhosis | Score cirrhosis if there is a positive biopsy and documented portal hypertension, OR  there have been previous periods of high gastrointestinal bleeding as a result of portal hypertension. | • | • |
| cardio_vasc_insuf | Angina or symptoms at rest or during minimal effort, such as dressing and personal hygiene (New York Heart Association class IV). | • | • |
| neoplasm | Metastases which have been diagnosed by clinical examination or confirmed by a pathology report OR if there is Stage IV cancer. | • | • |
| hem_malign | Encompasses malignant lymphoma, acute leukaemia or multiple myeloma. | • | • |
| aids | The patient is HIV-positive and has clinical complications such as pneumocystis carinii pneumonia, Kaposi's sarcoma, lymphoma, tuberculosis of toxoplasma infection, OR de patient is HIV-positive and has CD4 < 200. | • | • |
| imm_insuf | long-term immunosuppressive therapy, OR corticosteroid use (both short-term high and long-term low dosages, for example more than 5 days 1 mg/kg prednisone or more than 20 days >= 0.1 mg/kg), OR active chemotherapy or radiotherapy in the past year, ORhad chemotherapy or radiotherapy for Hodgkin’s or non-Hodgkins lymphoma at any time for IC admission, OR documented humoral or cellular deficiencies OR Hydrea use | • | • |
| mech_ventil_0 | Use of a ventilator at the moment of IC admission or immediately (within 15 minutes) thereafter. | • | • |
| cpr | Score if the patient has had CPR (heart massage) during the 24 hours prior to IC admission. Defibrillation and/or cardioversion without heart massage do not apply as CPR. | • | • |
| gastro_bleed | Encompasses hematemesis and melaena | • | • |
| diabetes | The patient has a medication-dependent form of diabetes. This must have been diagnosed before the current IC admission. | • | • |
| re_adm | https://www.stichting-nice.nl/dd/#459 | • | • |
| ref_spec | The specialism responsible for the patient’s admission to the IC, coded in accordance with SIG specifications (LMR User Manual, section 7, list 4: Specialism code list). | • | • |
| adm_source | The location (origin) from where the patient came immediately prior to admission to the IC. | • | • |
| adm_type | Indication of admission type. | • | • |
| plan_adm | An IC admission which is known about (planned) before physical admission and which could have been delayed for 12 hours without risk. | • | • |
| los_pre | the duration of treatment on the nursing ward before the current ICU admission (during the same hospitalization period) | • | • |
| bmi | BMI (weight in kg/ (length in m x length in m)) | • | • |
| aantalchronisch | Number of chronic diseases. | • | • |
| golf | COVID surge in which the patient was admitted. | • | • |
| album_measured | Was serum albumin measured? | • | • |
| bili_measured | Was bilirubin measured? | • | • |
| laatste_ic_ontslag | *Last IC discharge* - COVID patients were followed up across the hospitals, so if they have been transferred from an ICU to the ICU of another hospital, we have merged those two records into one record. The last IC discharge is the date of discharge from the last IC in which the patient was admitted. |  | • |
| laatste_zkh_ontslag | *Last hospital discharge* - COVID patients were followed up across the hospitals, so if they have been transferred from an ICU to the ICU of another hospital, we have merged those two records into one record. The last hospital discharge is the date of discharge from the last hospital in which the patient was admitted. |  | • |
| mech_ventil_24 | Use of a ventilator at any time during the first 24 hours of admission to IC. |  | • |
| ac_ren_fail | If there is: Renal replacement therapy at some point within the first 24 hours of ICU admission, or Serum creatinine level greater than 1.5 mg/100 ml (or 133µmol/l) during the previous 24 hours, associated with oliguria. |  | • |
| confirm_infection | Confirmed infection upon admission, or if infection is confirmed during the first 24 hours of IC treatment. |  | • |
| vasdrug | Continuous intravenous vasoactive medication for a minimum period of one hour during the first 24 hours of IC admission. |  | • |
| nice_ap4_excluded | The APACHE IV model has several inclusion criteria that an admission must meet to calculate a valid APACHE IV mortality probability. If excluded is set to 1, the patient in question does not meet the inclusion criteria of the model and is therefore excluded. |  | • |
| nice_ap4_score | APACHE IV score. |  | • |
| nice_ap4_prob | Hospital mortality risk according to the APACHE IV model. |  | • |
| verschillende_ics | *Number of different ICUs* - COVID patients were followed up across the hospitals, so if they have been transferred from an ICU to the ICU of another hospital, we have merged those two records into 1 record. The number of different ICUs shows on how many different ICUs the patient was admitted during the same hospitalization period |  | • |
| verschillende_zkh | *Number of different hospitals* – COVID patients were followed up across the hospitals, so if they have been transferred from an ICU to the ICU of another hospital, we have merged those two records into 1 record. The number of different hospitals shows to how many different hospitals the patient was admitted during the same hospitalization period. |  | • |
| beademingsduur_uren | How long the patient has been mechanically ventilated (in hours) during his entire ICU stay. |  | • |
| eye_low | Eye response at the time of the lowest GCS score during the first 24 hours of IC admission. |  | • |
| motor_low | Motor response at the time of the lowest GCS score during the first 24 hours of IC admission. |  | • |
| verbal_low | Verbal response at the time of the lowest GCS score during the first 24 hours of IC admission. |  | • |
| heartrate_min | Lowest heart rate during first 24 hours of IC admission. |  | • |
| heartrate_max | Highest heart rate during first 24 hours of IC admission. |  | • |
| resprate_min | Lowest respiratory rate during first 24 hours of IC admission (spontaneous or mechanical). |  | • |
| resprate_max | Highest respiratory rate during first 24 hours of IC admission (spontaneous or mechanical). |  | • |
| syst_min | Lowest systolic blood pressure during first 24 hours of IC admission. |  | • |
| syst_max | Highest systolic blood pressure during first 24 hours of IC admission. |  | • |
| meanbl_min | Lowest average blood pressure during first 24 hours of IC admission. |  | • |
| meanbl_max | Highest average blood pressure during first 24 hours of IC admission. |  | • |
| temp_min | Lowest rectal temperature during first 24 hours of IC admission. |  | • |
| temp_max | Highest rectal temperature during first 24 hours of IC admission. |  | • |
| urine_24 | The total urine output during first 24 hours of IC admission. |  | • |
| paco2 | Arterial CO2. Give the PaCO2 from the sample resulting in the highest alveolar-arterial oxygen pressure difference: (A-aDO2= 7.13*FIO2 - PaO2 - PaCO2) during the first 24 hours of IC admission. |  | • |
| fio2 | Percentage of oxygen inhaled. Give FiO2 relating to the sample with PaO2 and PaCO2 measurement which, when combined, result in the highest alveolar-arterial oxygen pressure difference in the first 24 hours of IC admission> The difference is calculated using the following formula: A-aDO2 = 7.13*FIO2 - PaO2 - PaCO2. |  | • |
| nice_pao2_fio2 | If the patient is ventilated or receives CPAP (continuous positive airway pressure), use the lowest value of the ratio between PaO2 and FiO2 in the first 24 hours of IC admission. |  | • |
| a_ado2 | Determine highest alveolar-arterial oxygen pressure difference: 7.13 * FIO2 - PaO2 -PaCO2. |  | • |
| pao2 | Arterial oxygen pressure. Give the PaO2 from the sample which results in the highest alveolar-arterial oxygen pressure difference (A-aDO2= 7.13*FIO2 - PaO2 - PaCO2) during the first 24 hours of IC admission. |  | • |
| ph_min | Value of arterial pH, measured in the same blood sample as for the measurement of oxygenation. |  | • |
| wbc_min | The lowest white blood cell (leukocytes) counts during the first 24 hours of IC admission. |  | • |
| wbc_max | The highest white blood cell (leukocytes) counts during the first 24 hours of IC admission. |  | • |
| creat_min | The lowest level of serum creatinine during the first 24 hours of IC admission. |  | • |
| creat_max | The highest level of serum creatinine during the first 24 hours of IC admission. |  | • |
| potas_min | The lowest level of serum potassium during the first 24 hours of IC admission. |  | • |
| potas_max | The highest level of serum potassium during the first 24 hours of IC admission. |  | • |
| sodium_min | The lowest level of serum sodium during the first 24 hours of IC admission. |  | • |
| sodium_max | The highest level of serum sodium during the first 24 hours of IC admission. |  | • |
| bicarb_min | The lowest level of serum bicarbonate during the first 24 hours of IC admission. |  | • |
| bicarb_max | The highest level of serum bicarbonate during the first 24 hours of IC admission. |  | • |
| urea | The highest level of serum ureum during the first 24 hours of IC admission. |  | • |
| bili | The highest level of total bilirubin during the first 24 hours of IC admission. |  | • |
| ht_min | The lowest level of hematocrit during the first 24 hours of IC admission. |  | • |
| ht_max | The highest level of hematocrit during the first 24 hours of IC admission. |  | • |
| hb_min | The lowest measured amount of hemoglobin in the blood during the first 24 hours of IC admission. |  | • |
| hb_max | The highest measured amount of hemoglobin in the blood during the first 24 hours of IC admission. |  | • |
| album_min | The lowest level of serum albumin during the first 24 hours of IC admission. |  | • |
| album_max | The highest level of serum albumin during the first 24 hours of IC admission. |  | • |
| throm_min | The lowest level of thrombocytes during the first 24 hours of IC admission. |  | • |
| gluc_min | The lowest level of serum glucose during the first 24 hours of IC admission. |  | • |
| gluc_max | The highest level of serum glucose during the first 24 hours of IC admission. |  | • |
| nice_ap4_aps | APACHE IV acute physiology score (APS). In other words, the physiological disruption in the first 24 hours of ICU admission. |  | • |
| nice_a_ado2 | The highest alveolar-arterial oxygen pressure difference in the first 24-hours of ICU admission. |  | • |
| nice_gcs_low | The lowest Glasgow Coma Score determined during the first 24 hours of IC admission. |  | • |
| hosp_discharged_to | Location of discharge after patients have been discharged from the hospital. |  | • |

**Supplementary Table s7.** Overview of predictors included in development of admission (0h) and 24h regression trees models. Further information can be obtained at <https://www.stichting-nice.nl/dd>.

**Supplementary Text s1. Working of decision trees.**

A decision tree algorithm learns recursively by iterating through the data and building a tree from the top node (root) to each of the leaf nodes. In each iteration, nodes may be split into two nodes (subgroups). The split is based on the value of a variable. For example, “age ≤ 78” splits a patient group into two subgroups: younger than or are 78 years and older than 78 years. The variables and values are automatically determined by the learning algorithm, as an attempt to reduce the average variance of the two generated subgroups. A node that no longer split is called a leaf. Each path from the starting point (whole study population) to a leaf represents a rule. Each leaf node corresponds to a subgroup of patients that fulfil the conditions of all branches from the root node leading up to the leaf node (i.e., compliant with the path rule).
