# Supplementary figures and images for: Development and evaluation of regression tree models for predicting in-hospital mortality of a national registry of COVID-19 patients over six pandemic surges
Source: BMC Med Inform Decis Mak. 2024 Jan 2;24:7. doi: 10.1186/s12911-023-02401-2 (PMC10762959; doi:10.1186/s12911-023-02401-2)

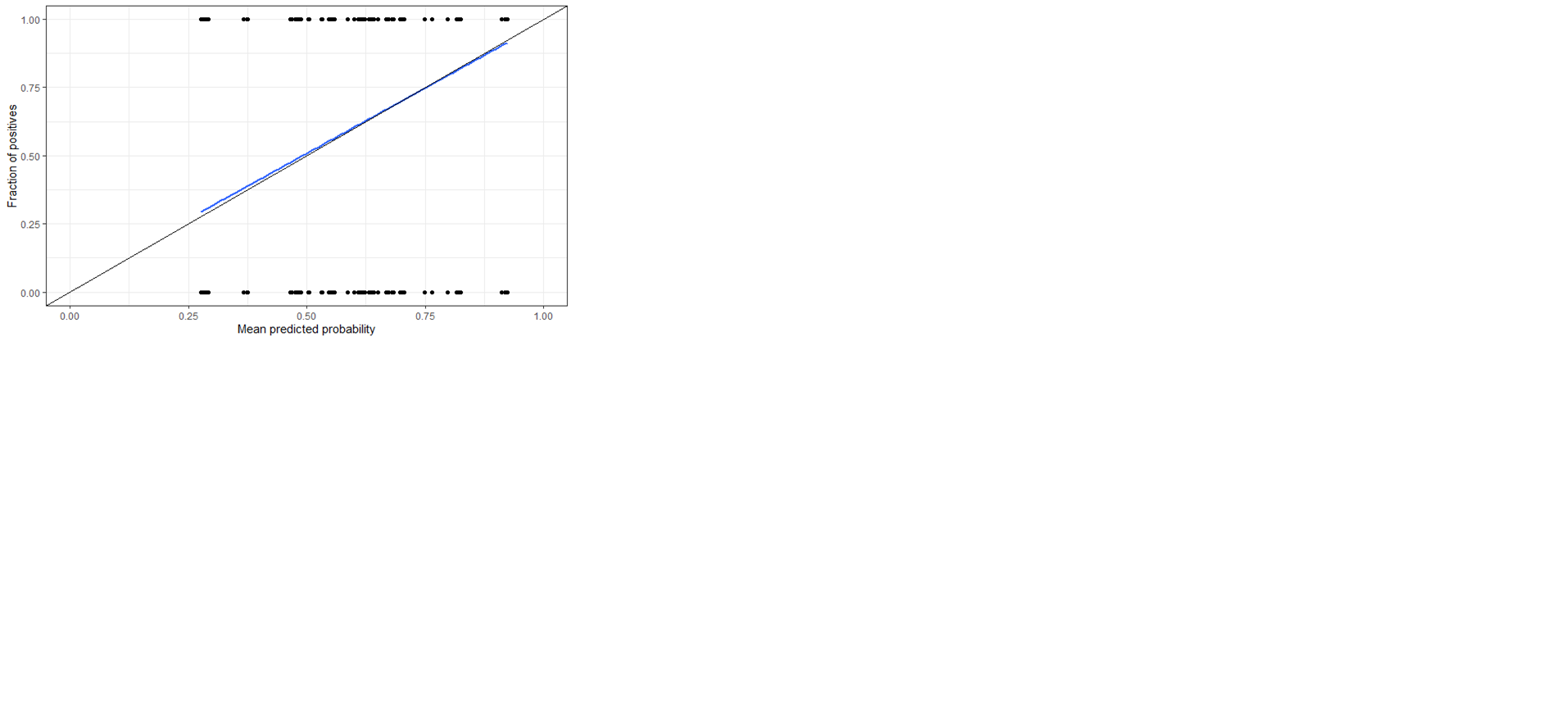

Supplement: Supplementary file 2 — Additional file 2. [file 12911_2023_2401_MOESM2_ESM.tif]
